# The importance of including aliases in data linkage with vulnerable populations

**DOI:** 10.1186/s12874-018-0536-4

**Published:** 2018-07-06

**Authors:** Holly Tibble, Hsei Di Law, Matthew J. Spittal, Rosemary Karmel, Rohan Borschmann, Katie Hail-Jares, Laura A. Thomas, Stuart A. Kinner

**Affiliations:** 1 Centre for Mental Health, Melbourne School of Population and Global Health, University of Melbourne, Melbourne, Australia; 2 Data Linkage Unit, Australian Institute of Health and Welfare, Canberra, Australia; 3 Centre for Adolescent Health, Murdoch Children’s Research Institute, Melbourne, Australia; 4 Health Service and Population Research Department, Institute of Psychiatry, Psychology and Neuroscience, King’s College London, London, UK; 5 Centre for Health Policy, Melbourne School of Population and Global Health, University of Melbourne, Melbourne, Australia; 6 Griffith Criminology Institute, Griffith University, Brisbane, Australia; 7 Mater Research Institute-UQ, University of Queensland, Brisbane, Australia; 8 School of Public Health and Preventive Medicine, Monash University, Melbourne, Australia; 9 Department of Psychiatry, University of Melbourne, Melbourne, Australia

**Keywords:** Data linkage, Probabilistic, Aliases, Vulnerable, Indigenous, Youth, Justice

## Abstract

**Background:**

Records pertaining to individuals whose identity cannot be verified with legal documentation may contain errors, or be incorrect by intention of the individual. Probabilistic data linkage, especially in vulnerable populations where the incidence of such records may be higher, must be considerate of the usage of these records.

**Methods:**

A data linkage was conducted between Queensland Youth Justice records and the Australian National Death Index. Links were assessed to determine how often they were made using the unverified (alias) records that would not have been made in their absence (i.e. links that were not also made using solely verified records). Anomalies in the linked records were investigated in order to make evaluations of the sensitivity and specificity of the linkage, compared to the links made using only verified records.

**Results:**

From links made using verified records only, 1309 deaths were identified (2.6% of individuals). Using alias records in addition, the number of links increased by 16%. Links made using alias records only were more common in females, and those born after 1985. Different records belonging to the same individual in the justice dataset did not link to different death records, however there were instances of the same death record linking to multiple cohort individuals.

**Conclusions:**

The inclusion of aliases in data linkage in youths involved in the justice system increased mortality ascertainment without any discernible increase in false positive matches. We therefore conclude that alias records should be included in data linkage procedures in order to avoid biased attenuation of ascertainment in vulnerable populations, leading to the concealment of health inequality.

## Background

Probabilistic data linkage does not require perfect matching for a link between two datasets to be established. Rather, record pairs are created by matching exactly on selected variables (if desired) and allowing remaining variables to vary. One aspect of data linkage that is often not considered when matching data from the general population, but which may be particularly relevant for vulnerable populations such as people with a history of incarceration, is the use of aliases - defined herein as any record for an individual in which demographic variables differ from ‘birth’ or ‘legal’ records verified with documentation.

In the general population, aliases may arise due to any number of reasons, including errors in the recording or entering of original data, cultural and tribal (such as Aboriginal) names, nicknames, and preferred names. In the criminal justice setting, intentionally concealing or obscuring one’s identity is also common [1, 2]. Increased incidence of aliases is found across many vulnerable populations, however, when handled correctly, aliases can be utilised to enhance linkage quality using probabilistic methods [3–5]. This is especially relevant in the estimation of standardised mortality ratios and other mortality metrics, where unidentified deaths may introduce an attenuating bias [5–7].

We set out to compare the quality of linkage between youth justice and mortality records, with and without the inclusion of aliases, among young people with a history of contact with the youth justice system in the state of Queensland, Australia. We hypothesised that the use of additional link identifiers/aliases would result in more true matches, without a proportional increase in false matches.

## Methods

### Study design and participants

The study cohort consisted of all young people who were either detained, sentenced to a community-based order, or charged with an offence in the state of Queensland between 1 January 1993 and 31 December 2014. This comprised 94,921 records, representing 51,263 young people.

### Data sources

Youth Justice Queensland provided cohort data including name, sex, date of birth, and date of last contact with Youth Justice Queensland. An additional variable indicated the type of record that was supplied: ‘birth’, ‘legal’ (legally documented name changes, for example after marriage or divorce, or due to personal choice) or ‘alias’. The inclusion of a (non-re-identifiable) unique identifier allowed us to determine which birth, legal and alias records belonged to the same individual.

Death records were extracted from the National Death Index (NDI), a Commonwealth database containing records of all deaths registered in Australia since 1980, and included name, sex, date of birth, and state of death registration (approximately five million records). Deaths up to 31 January 2017 were included in the NDI extract used for this study.

Access to the identifying information in the datasets was not available to authors HT, KHJ, MS, RB, LT or SK - only to HDL and RK, at the Australian Institute of Health and Welfare (AIHW), who facilitated the data linkage.

### Data linkage

In probabilistic linkage, records are paired by matching exactly on selected variables and allowing remaining variables to vary. A weight is assigned to each potential record pair to determine its strength – based on the ratio of the probability of a match being true to the probability that records will match at random, for a given set of values for the specified linkage variables [8]. In the current application the weights were derived using the standard Fellegi-Sunter probabilistic procedure [9] within DALI, the AIHW’s linkage system.

It was considered a priori that the use of additional link identifiers/aliases was likely to result in more true matches. Given the relatively small size of the cohort data set and the high quality of data in both the cohort and NDI data sets, full manual review of potential matches was performed to determine matches in both scenarios, instead of solely those between chosen weight thresholds.

In this data linkage, all records (whether birth, legal or alias) for the same individual were treated equally and allowed to match independently; that is, record status was not considered when identifying matches. It was then determined for each match whether the link was ascertained using a verified record (either birth or legal), and/or an alias record. As such, the matches identified using verified records were not altered by the inclusion of alias records in the procedure.

Some links were identified using either the verified records alone, or both verified and alias records; in which case the alias records were superfluous. We refer to these links as “verified links” and links identified using the alias records only as “alias links”.

### Data analysis

Sensitivity of the linkages was calculated by comparing the linkage conducted with all records (the hypothesised gold standard) to the linkage without aliases – treating matches identified using alias records only as false negatives.

Links were also assessed to determine unidentified duplicates in either dataset. For example, in the instance that a mortality record linked to multiple YJ records, we ascertained whether the YJ records all belonged to the same individual. Links were also assessed further if there was a date of contact that followed supposed death, in order to inform judgements about whether the link was a false positive. This in turn informed judgements about the linkage accuracy, as a pseudo-specificity measure.

We also examined differences in rates of alias links by sex, and year of birth (dichotomised at 1985 to most evenly divide the cohort).

Even though the data come from the population of young people who have had contact with the youth justice system, we use inferential methods to test for differences between groups on the basis that the data are a sample of the population from a single jurisdiction and at a particular period in time.

## Results

The majority of records had recorded the individual’s name as a ‘birth’ name (52.7%) or ‘legal’ name (2.1%); 45.2% of records were alias records. Over half of individuals had only one record (28,859; 56.3%); in 28,127 (97.5%) cases, this was a ‘birth’ record. The remaining 22,404 (44.7%) individuals had multiple records, with 5343 (10.4%) having four or more records (up to a maximum of 22 records per individual). Name variation was several times more common than date of birth variation (29.9, 16.7 and 9.1% of individuals had variation of surname, first name, and date of birth, respectively).

Multiple records were found to be more common for females: 6856 (54.5%) females had multiple records with an average of 3.3 non-birth records (amongst those with multiple records; 95%CI: 3.2–3.3); 15,489 (40.2%) males had multiple records, with an average of 2.8 non-birth records (95%CI: 2.8–2.8).

A total of 1518 mortality records were linked to individuals in the cohort. Fourteen mortality records were each linked to two (unique) identifiers. Clerical review by AIHW suggested that in each case these were for the same individuals with two person IDs (duplicate ID numbers) in the cohort data set (implying 1504 deceased individuals). Because duplicates linkages may be an indicator of linkage quality, they were retained for the purposes of this study.

**Fig. 1:**
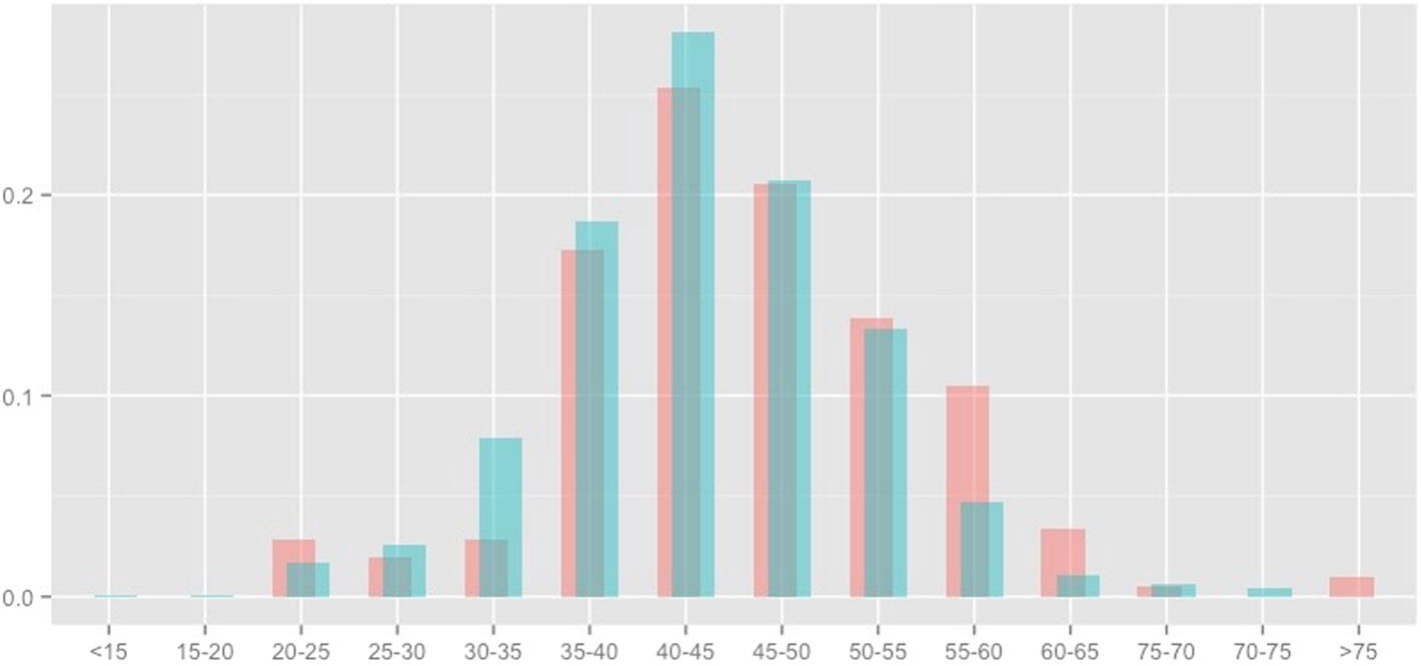
“Density plot of match weights by record type”. Vertical Axis Title: “Density”. Horizontal Axis Title: “Linkage Weight”. Blue = “Verified Record Matches”, pink = “Alias Record Matches”

Using verified links only, 1309 deaths were identified (2.6% of individuals). An additional 209 deaths were identified (total 1518, 3.0%) via alias links, so that the number of links increased by 16.0% (*p* < 0.001). Taking the linkage using both verified and alias records as the gold standard leads to a sensitivity estimate of 86.2% (95% CI: 84.4–87.9) for the verified links – meaning that 13.8% of deaths would have been missed (false negatives) if alias records had been ignored. Matches using verified records had a slightly lower average weight than those using alias records (Fig. 1).

For males, 88% of identified links were made without the use of alias records, compared to only 76% in females (chi-squared *p*-value < 0.001). In those born before 1985, 12% of links were made using alias records alone, compared to 21% in those born in 1985 or later (chi-squared *p*-value < 0.001).

Of the identified deaths, 69 (4.5%) had a date of last contact with Youth Justice Queensland that was after their recorded death date. All 69 cases had exact matches on name and sex between the datasets; however, six of these individuals did not have an exact birth date match; these six matched on year of birth only, with day and month of birth missing in the linked NDI records. 12 of the 69 matches (17%) were made using alias information only.

Assuming that the dates of Youth Justice contact were correct and all 69 cases were false positives (a maximally conservative assumption), in addition to the 28 cases linked to 14 death records, we found that the rate of false positives was similar (chi-squared *p*-value: 0.914) among alias only (13/209; 6.2%) and verified matches (84/1309; 6.4%).

## Discussion

### Summary of results

Our findings indicate that 13.8% of deaths in the cohort would have been missed had alias records not been included in the linkage. When an individual was matched using both verified and alias records, the alias records did not identify any links to death records which differed from that made using the individual’s birth records, suggesting that inclusion of aliases increased sensitivity without adversely affecting specificity.

### Results in context

Links made using unverified records only were found to be twice as common in females as in males (24% vs 12%). Unverified records may include legal name changes (for example, following marriage) which could not be verified using documentation at the time of the record entry or for subsequent episodes of contact with youth justice. Higher prevalence of non-birth records in females may therefore be due to name changes after marriage, however, we also note that while surname variations were more common in females (39.6% vs 26.7%), so were first name variations (24.9% vs 14.0%). This implies that better access to verified name records would be insufficient to eliminate gender imbalance in data linkage.

Inclusion of alias records is critical to maximising ascertainment, particularly in populations where their usage is more common, such as those exposed to the criminal justice system. Failure to include these records can result in disproportionate under-ascertainment in vulnerable populations, for whom targeted cohort studies tend to be expensive and underpowered [10].

Our findings are an important addition to this body of knowledge. We found that the inclusion of aliases in data linkage increased the number of links without contradicting the links found with verified records only. This is concordant with a 2011 Australian study, linking adult correctional records to mortality records [5], which found that including aliases as a linkage identifier increased the sensitivity of matching from 64 to 86%.

Given the increasing reliance on administrative data to inform government policy and service provision [11], it is essential that we continue to identify areas in which systematic bias may affect data linkage [12, 13], particularly in vulnerable and hard-to-reach populations, for whom data linkage studies are often the primary research base.

### Strengths and limitations

Although previous studies have assessed the utility of aliases in data linkage with incarcerated adults, this is, to the best of our knowledge, the first study to assess data linkage quality using aliases in a youth justice population. Based on these findings, we suggest that including aliases in data matching may also have relevance to other vulnerable and marginalised populations.

Our study had two main limitations. First, our cohort data came from one Australian state, and although it is likely that our findings would be applicable elsewhere, we are unable to assess this. Second, without knowledge of the true classification (in this case, dead or alive) we were unable to independently verify the sensitivity and specificity of the linkage. As such, we were reliant on proxy measures, which may overstate the sensitivity of the linkage (ability to detect false positive links). In an attempt to counter this, we were conservative in our critique of anomalies such as dates of contact after supposed death.

While a full manual review of all record pairs would not be plausible for all linkages, using a lower threshold would not change the finding that using unverified records in addition to verified records improves results.

## Conclusions

The inclusion of aliases in data linkage with justice-involved youths increased ascertainment of mortality by 16%. Consistency checks indicate that, although we had no independent gold standard against which to compare, using aliases is not likely to have increased the false positive rate. Inclusion of aliases in data linkage augments linkage quality, and avoids biased attenuation of ascertainment that could mask health disparities in vulnerable populations.

## Abbreviations

AIHW: Australian Institute of Health and Welfare
HREC: Human Research Ethics Committee
NDI: National Death Index
